# Biofilm of *Helicobacter pylori*: Life Cycle, Features, and Treatment Options

**DOI:** 10.3390/antibiotics12081260

**Published:** 2023-07-31

**Authors:** Yasmine Elshenawi, Shuai Hu, Skander Hathroubi

**Affiliations:** 1 Department of Microbiology and Environmental Toxicology, University of California, Santa Cruz, CA 95064, USA; yelshena@ucsc.edu; 2 Spartha Medical, CRBS 1 Rue Eugène Boeckel, 67000 Strasbourg, France

**Keywords:** *Helicobacter pylori*, biofilms, planktonic, antibiotic resistance, extra polymeric substance, abiotic/biotic adhesion, dispersion, clinical treatment strategies, anti-biofilm strategies

## Abstract

*Helicobacter pylori* is a gastric pathogen that infects nearly half of the global population and is recognized as a group 1 carcinogen by the Word Health Organization. The global rise in antibiotic resistance has increased clinical challenges in treating *H. pylori* infections. Biofilm growth has been proposed to contribute to *H. pylori’s chronic* colonization of the host stomach, treatment failures, and the eventual development of gastric diseases. Several components of *H. pylori* have been identified to promote biofilm growth, and several of these may also facilitate antibiotic tolerance, including the extracellular matrix, outer membrane proteins, shifted morphology, modulated metabolism, efflux pumps, and virulence factors. Recent developments in therapeutic approaches targeting *H. pylori* biofilm have shown that synthetic compounds, such as small molecule drugs and plant-derived compounds, are effective at eradicating *H. pylori* biofilms. These combined topics highlight the necessity for biofilm-based research in *H. pylori*, to improve current *H. pylori*-targeted therapeutic approaches and alleviate relative public health burden. In this review we discuss recent discoveries that have decoded the life cycle of *H. pylori* biofilms and current biofilm-targeted treatment strategies.

## 1. Introduction

*Helicobacter pylori* is a Gram-negative, spiral-shaped, bacterial pathogen that colonizes the gastric epithelium [1,2,3]. *H. pylori* has been globally recognized as a high priority pathogen as it has been associated with various gastric diseases, including peptic ulcers, chronic gastritis [4,5], gastric mucosa-associated tissue lymphomas [6], and gastric adenocarcinomas [7,8,9,10]. Mechanisms of transmission remain unknown [10], but antibiotic therapies used to treat *H. pylori* infection have alarmingly been losing efficacy in regions with high infection burden [11]. Antibiotic-resistant *H. pylori* was reported to disproportionately affect children in Asian, African, and European countries [12], and in underserved communities in the US [13]. One current perspective is that *H. pylori* in biofilms, a low growth state, may substantially promote antibiotic resistance and persistence in the host stomach [14]. *H. pylori* were initially observed in vitro to form water-insoluble biofilms which are defined as stationary aggregates of cells encased in extra polymeric substances (EPS) [15,16]. *H. pylori* with biofilm state have also been observed in the gastric mucosa of patients with peptic ulcers [17,18]. In this review, we discuss recent discoveries that characterize the features, decode regulation processes of *H. pylori* biofilm growth in vitro and in vivo, elucidate supportive evidence of antibiotic tolerance and current developing biofilm-targeted anti-*H. pylori* strategies.

## 2. General Features of *H. pylori* Biofilms

*H. pylori* biofilms consist of stationary aggregates of cells encased by an extracellular matrix composed of proteins [19], extracellular DNA [20], and polysaccharides [21]. *H. pylori* biofilm formation starts from planktonic cells that adhere to either abiotic or biotic surfaces, leading to the formation of microcolonies with three-dimensional structures [22,23]. Additionally, *H. pylori* cells can cluster together as non-surface-attached aggregates, a form that has been recently observed and recognized as a biofilm format in other bacterial studies [24]. Once adhered, *H. pylori* biofilm formation was found to occur optimally under conditions lacking nutrients, such as fetal bovine serum [25,26].

**Scheme 1:**
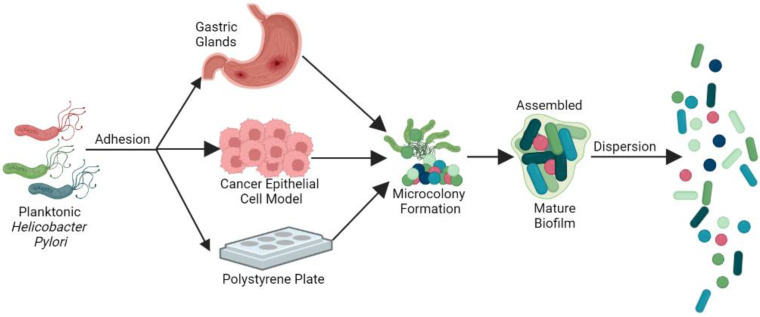
*Helicobacter pylori* Biofilm Lifecycle. *H. pylori* adheres to both abiotic and biotic surfaces, where it forms microcolonies that susbquently assemble into mature biofilms characterized by the presence of extracellular polymeric susbtances (EPSs). Dispersion allows bacteria to colonizes new niches.

Aside from biofilm growth on abiotic surfaces, additional studies have also suggested that *H. pylori* can form a microcolony network that adhered and grew between epithelial cell junctions on human cells [27,28] and in murine gastric glands [29]. Mature *H. pylori* biofilms consist of different cell shapes within one multicellular population. For example, both spiral and coccoid *H. pylori* cells were simultaneously observed from one gastric biopsy [17]. Similarly, on abiotic surfaces, most cells adopt the coccoid morphology, with the minority displaying a rod shape [30,31]. As found in other bacteria, *H. pylori* biofilm formation exhibits a similar multiple-step process, including bacterial adherence, biofilm assembly, mature biofilm formation, and dispersion (Scheme 1). In the next sections, we dissect the features of each step in *H. pylori* biofilm growth.

## 3. Adherence

Adhesion is an essential process that initiates *H. pylori* biofilm formation and retains a role throughout the lifetime of the biofilm [32,33,34]. Prior studies have found that *H. pylori* can adhere to both gastric epithelial cells [35] and abiotic surfaces [32]. *H. pylori* surface adhesion and microcolony formation was first negatively associated with the concentration of supplemented fetal bovine serum (FBS); serum commonly promotes planktonic growth but inhibits surfaces adherence [26]. It remains elusive which factors of serum impact *H. pylori* surface adhesion as FBS is an undefined medium with a non-homogeneous mix of growth factors [36]. Interestingly, *H. pylori* adhesion on gastric epithelial cell surface does not rely on the presence of FBS, suggesting that this bacterium may utilize a specialized mechanism for surface attachment [22,37,38,39]. Furthermore, studies have shown that adhesion on various surfaces directly affected the biomass of mature biofilms, a process that is independent of media components [19]. *H. pylori* surface adhesion is also strain-dependent [34], a variation that is potentially due to the heterogenicity of regulatory proteins and outer membrane proteins (OMPs) which are predicted to play a critical role in the initial adhesion step [34,40]. We focus on discussing recent findings that have implicated flagella and OMPs as necessary components in the adherence process.

*H. pylori* flagella play important roles in adherence and subsequent biofilm formation. They are made up of four primary components; basal body, hook, filament, and sheath [41]. Flagella are typically associated with *H. pylori* motility but have been recently discovered to be involved in promoting surface adhesion and maintaining biofilm architecture [23]. Motility itself is an essential factor required for *H. pylori* to initiate biofilm [34]. More insight was provided for this observation by examining abiotic biofilm formation of strains that were non-motile but either retained flagella (Fla^+^ Mot^−^) due to deletion of a flagellar basal body gene *motB*, or lost flagella (Fla^−^ Mot^−^) due to deletion of the flagellar basal body gene *fliM* [25]. More biofilm biomass was accumulated in the Fla+ mutant compared with the Fla- strain. Flagella filaments, furthermore, were visible in the biofilm, and appeared to be forming a matrix. Fla^+^ Mot^−^ strains exhibited initial attachment defects on gastric cell surfaces [23]. These results suggest that motility is likely involved in the attachment phase on diverse surfaces, and the presence of flagella is required for *H. pylori* biofilm formation.

Another type of molecule shown to contribute to adherence are *H. pylori* outer membrane proteins (OMPs), which can be on the cell surface or present in outer membrane vesicles (OMVs) along with virulence factors and eDNA [42,43]. OMPs play important roles in bacterial environmental adaptation and modulation of life cycle phases, including structure maintenance, substance transportation, and microbial–host interaction [44,45].

*H. pylori* has more than 60 OMPs coding genes [46], but not all the OMP’s functions are understood [40]. Beyond inducing the pro-inflammatory responses, some OMPs were also found to promote multiple processes of *H. pylori* biofilm formation, one of which is to promote surface adhesion as discussed [47,48]. *H. pylori* OMPs facilitate both cell-to-cell and cell-to-abiotic surface adhesion in biofilms, based on the observation of OMV localization in *H. pylori* biofilms via scanning electron microscopy (SEM) imaging [49].

A common feature of *H. pylori* OMPs is anti-parallel β sheets that compose a β-barrel, highly stable pore-like structure; transmembrane domains of these proteins interact with host cell receptors [49], potentially indicating these OMPs may promote bacterial cell–cell and bacterial–host connections.

One family of *H. pylori* OMPs is the Hom family, a group of four proteins encoded by the following genes: *homA*, *homB*, *homC*, and *homD* [46]. These proteins have been specifically utilized as a peptic ulcer disease marker [49]. Interestingly, *homA* and *homB* were found to contribute *H. pylori* biofilm formation as well [50], indicating the potential association between *H. pylori* biofilm and relative pathology. The outer membrane protein *homB* (J99, *jhp0870;* G27 *HPG27_667*), was recently associated with biofilm formation [40]. This protein is interesting as it has been proposed as a biomarker of peptic ulcer disease [51] and gastric cancer [52]. *H. pylori* upregulates *homB* transcription via ArsRS, a two-component system, in the initial adherence and assembly phases of biofilm growth, but then levels fall back to those observed in planktonic cells after 72 h of incubation [50]. This variation suggests the importance of HomB during the initial adhesion and later for the next biofilm assembly stages.

HomA and HomB are composed mainly of β-sheets with cysteine resides on surface loops that help to form homodimers and indicate that they are potentially key to aggregation and biofilm formation [48]. Various studies demonstrated that HomB is negatively regulated by a two-component system, ArsRS system [19,25,50]. Other Hom family members, the *homD* and *homC* genes, are both upregulated during *H. pylori* biofilm formation [25]. Polymorphism of HomC have been linked to varied levels of biofilm formation in different *H. pylori* strains [53]. These findings suggest Hom family OMPs are commonly involved in the initiation steps of biofilm formation.

The outer membrane protein autotransporter is also likely to play a role in regulating *H. pylori* biofilm formation. An uncharacterized autotransporter, paralogous to VacA, *vlpC*, was found to cause a defect of *H. pylori* biofilm formation if disrupted [23,46]. Specifically, this mutant was unable to form mature biofilms. *vlpC* has been upregulated in some biofilms, further supporting this factor is important for *H. pylori* biofilm formation [23,25].

A group of highly conserved laminin binding proteins of another OMP family, called Hop, has also been shown to be involved in *H. pylori* biofilm regulation as well. AlpB, a Hop family member, was implicated in biofilm formation and antibiotic resistance, since the genetic deletion of *alpB* caused less *H. pylori* biofilm formation [37,38]. Since AlpB is highly conserved among *H. pylori* strains, it has recently been identified and investigated as a therapeutic target to eradicate *H. pylori* biofilm [54].

These findings highlight the role played by OMPs and flagella at this stage, while also emphasizing that there is much to be discovered.

## 4. Assembly

After surface adhesion, *H. pylori* starts forming microcolonies or aggregates that are recognized as the pre-mature form of biofilm [23,25,55]. Multicellular aggregates have been observed to be formed by different strains in in vitro conditions within hours [56], and more complex structures as early as one day of incubation [23,25]. *H. pylori* biofilms formation steps have been characterized using confocal microscopy. This work showed that *H. pylori* strain G27 assembles biofilms initially at the liquid air interface at 24 h, then assembles aggregates both at the liquid air interface and under the liquid air interface as the biofilm assembled; the distribution of EPS, visualized by staining, paralleled this growth trend [19]. SEM further revealed that flagella play a critical role in maintaining *H. pylori* biofilm structure, as discussed above [23,25]. Without flagella, *H. pylori* biofilms were slowly assembled [33]. Comparative genomics studies further demonstrated *H. pylori* biofilm assemble at rates that are similar among different strains when calculating cumulative frequency and rate of formation [34]. Additionally, biofilm assembly is not significantly impacted by in vitro conditions, such as serial passaging, nutrient compositions, and culturing conditions [19].

## 5. Mature Phase

The maximum biofilm mass can be observed after 3 days in vitro incubation [23,57], and can last up to 7 days in different culture conditions [19,31,33,34]. Comparing biofilm growth on the surface of polystyrene plates (hydrophobic surface) that were pre-coated with poly-D-lysine (hydrophilic and positive charged) and tissues culture treated polystyrene (hydrophilic, negative charge) revealed that optimal biofilm growth is not solely dependent on surfaces being ionic; tissue culture treated and negatively charged surfaces significantly promotes biofilm growth [19]. A special feature of *H. pylori* biofilms observed in SEM images are flagellar filaments which were discovered to promote surface cohesion and cell-to-cell connections as mentioned above, together with pili formations sustain the biofilm structural integrity on both abiotic and biotic surfaces [23,25].

In the meantime, different *H. pylori* strains and incubation conditions can differentially impact biofilm formation kinetics. *H. pylori* strains with strong and poor biofilm-forming abilities in tissue culture plates had consistent biomass accumulation rates during the intermediate assembly phases but had a variant cumulative biomass at the mature phase after 7 days of growth [34]. In another study, *H. pylori* SS1 strain produced robust biofilms in relatively low FBS conditions after 3 days of growth on polystyrene plates, with most biofilm cells (~80%) being coccoid shaped [25]. Interestingly, *H. pylori* G27 strain did not rely on low-serum conditions, as biofilm formation was not impaired even at standard culture media, with 10% FBS and produced biofilms with similar morphological features as SS1 [23].

**Figure 1:**
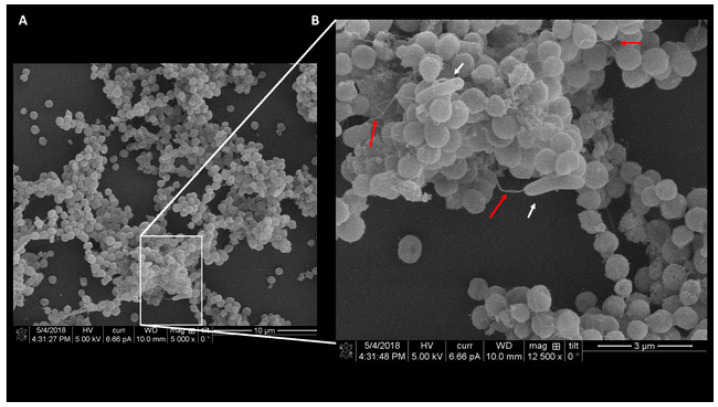
SEM images of mature *H. pylori* biofilms grown on abiotic surfaces. (**A**) Mature *H. pylori* biofilms contain a mixed population of mostly cocoid-shaped cells with a minority of spiral-shaped cells, (**B**) higher resolution image showing spiral-shaped cells (white arrow) and coccoid cells aggregating via the flagella (red arrow).

In mature biofilms grown on abiotic surfaces (Figure 1), most cells are coccoid-shaped (0.4–0.6 um long) with a minority of rod-bacillus (2–3 um long) shape [23]. The coccoid form of *H. pylori* was proposed as a response to the environmental stressors, but the underlying mechanism for this morphology is not fully characterized [58]. A recent study showed that these coccoid cells maintained their membrane integrity and metabolism for up to 70 h of incubation, which strongly suggests that they are viable dormant bacteria [59]. A morpho-structural analysis of *H. pylori* biofilms revealed that the strongest biofilm-producing cells show a dominance of coccoid forms unlike weak biofilm-producing cells which presented rod-shaped forms that were dominant in mature biofilms [60].

Interestingly, *H. pylori* in the coccoid morphology is more tolerant to antibiotic exposure [56] which aligns with *H. pylori* biofilm’s strong tolerance to antibiotics [23,25]. Viability staining experiments with biofilms grown on abiotic surfaces suggest that live cells and dead cells compose matured *H. pylori* biofilms [23]. Transcriptomic experiments show that biofilm cells are less metabolically active than planktonic cells due to the downregulation of multiple metabolic genes, such as *atpC*, *atpE*, and *nifU* [25]. Gastric epithelial cell lines, such as AGS, have been developed to study *H. pylori* biofilm formation on biotic surfaces [23,49]. After co-incubating *H. pylori* and AGS cells for days, *H. pylori* biofilms were observed on the surfaces and between conjunctions AGS cells [23,61]. Interestingly, most biofilm cells were spiral/rod-shaped, a different outcome than what was observed in biofilms grown on abiotic surfaces [23]. Other cell lines have been employed as well, particularly mucin-producing cells, like MKN-45 cells, which may present a more natural in vivo-like state similar to niches in the host. On the MKN-45 cell line, most of the biofilm cells primarily exhibited the coccoid morphology [62], suggesting this cell line can be used as a model to study the effects of mucin on *H. pylori* biofilm formation. Further studies are necessary to dissect whether different incubation conditions, such as serum concentration and incubation period may modulate *H. pylori* biofilm features.

## 6. Dispersion

Like other bacterial biofilms, *H. pylori* biofilms disperse after reaching optimal growth, indicated by a decrease in crystal violet staining after maximum growth has been reached [23,25,56]. Little is known about the signals that lead to *H. pylori* biofilm dispersal, but some evidence suggests that *H. pylori* utilizes a quorum-sensing molecule, AI-2, as a signaling molecule to regulate biofilm generation and dispersion [28]. AI-2 was initially recognized as a chemorepellent of *H. pylori* sensed by chemoreceptor TlpB [63], and this molecule can be expressed by *H. pylori* through *luxS* gene in a cell density-dependent manner [64,65], suggesting that *H. pylori* can efficiently control local density through AI-2 secretion. A later study suggested that AI-2 promoted *H. pylori* biofilm dispersion, as genetic deletion of the *luxS* in *H. pylori* significantly promoted its biofilm formation in comparison to isogeneic WT strain through the lacunarity and fractal dimension analysis [28]. The chemotaxis system, in another aspect, was suggested to facilitate *H. pylori* biofilm dispersion by sensing and responding to AI-2, since chemotactic histidine kinase-deficient mutant *∆cheA* exhibited similar biofilm phenotype as the *∆luxS* mutant [28]. Further research is required to decipher the mechanism of how *H. pylori* regulates biofilm maturation and dispersion.

## 7. *H. pylori* Clinical Treatment Strategies Become Less Efficient, Highlighting the Requirement of Alternative Strategies

Due to the persistence of disease development in *H. pylori* infections that has been exacerbated due to the COVID-19 pandemic, a recent consensus report states a need for consistent updates in clinical treatments, including effective testing and preventative measures for gastric illness [66]. Globally, different geographic regions have variable patterns of anti-microbial resistance [12], a component which should be used to determine treatment strategies according to recent European [66], Chinese [67], and Canadian [68] consensus reports. A challenge to developing effective treatments strategies for these infections is the rising rate of antibiotic resistance and the diversity of clinical and symptom scenarios associated with *H. pylori* infections [66].

In regions with a high prevalence in *H. pylori* infection, current clinical guidelines recommend a quadruple therapy that consists of bismuth, proton pump inhibitor (PPI) or potassium-competitive acid blocker and two different antibiotics (i.e., including clarithromycin, metronidazole, levofloxacin, or amoxicillin) [12,66,67,69]. Non-bismuth quadruple therapies are also recommended and have the following components: PPI and three antibiotics [66,69]. However, this classical therapeutic strategy has been being less effective due to the continuing global rise of antibiotic resistance [70]. For example, in 2016 a national consensus on Chinese management of *H. pylori* infections where quadruple therapy is used reported that metronidazole, levofloxacin, and clarithromycin resistance was 40–70%, 20–50%, and 20–50%, respectively [67]. Similarly, the elevation of antibiotic resistance was also noticed in other countries, like Indonesia, that apply the triple therapy approach consisting of PPI and two antibiotics [71]. Metronidazole and levofloxacin, two commonly applied antibiotics, were observed to be resisted by 46.7% and 31.2% of *H. pylori*-infected population, respectively; while those less commonly applied antibiotics exhibited relative lower resistance prevalence, including amoxicillin (5.2%), tetracycline (2.6%), and clarithromycin (9.1%) [71]. In 2020, a case study reported that triple therapies in Indonesia were further decreased to only 67.6% efficient [72].Aside from Indonesia and China, alarming clarithromycin resistance rates are observed in the Americas (10%), the African region (15%), Eastern Mediterranean region (29%), Europe (32%) which is why the WHO has designated clarithromycin-resistant *H. pylori* as a high priority research pathogen [70].

A meta-analysis review based on global WHO regions reported that clarithromycin resistance decreased the efficacy empiric eradications to less than 80%; additionally, metronidazole resistance was observed in >27% strains and levofloxacin resistance in >14% strains from all surveyed WHO regions in 2018 [70]. To counter potential therapy failure caused by antibiotic resistance, clinicians have proposed using a tailored treatment approach based on antibiotics susceptibility tests and localized resistance [68,72,73,74,75]. A clinical study that analyzed the failure of treatment revealed that isolated *H. pylori* has either individually or populationally developed multidrug resistance [76]. A study genotyped 112 *H. pylori* strains isolated from a region with prevalent *H. pylori* infection that apply quadruple treatment found strains with dual resistance to metronidazole and levofloxacin (20.5%) and triple resistance to metronidazole, clarithromycin, and levofloxacin (~7%) [11]. A study investigating the tailored treatment strategy found that out of 40 patients, some patients were infected with multiple strains or singular strains that exhibited different resistance phenotypes depending on the region of stomach the strain was isolated from [76]. Clarithromycin resistance is attributed to mutations in the 23S rRNA [77]; metronidazole resistance was associated to the mutations in *rdxA* and *frxA* loci [78]; levofloxacin resistance was caused by *gyrA* and *gyrB* mutations [79]. These mutations are naturally occurring, but increased prevalence in the population can occur by exposing strains to sub-MIC levels of antibiotics, such as levofloxacin [80]. To address these resistance-based challenges, a clinical trial evaluated the effectiveness of tailored therapies in comparison with the traditional bismuth quadruple therapy, and it was demonstrated that the tailored bismuth/quadruple therapy was more effective [68]. Intriguingly, another case study examined 101 clinical *H. pylori* isolates from Indonesian patients with gastritis (90.1%), peptic ulcer disease (8.9%), and gastric cancer (1%) and discovered that 93% of the isolates formed biofilms [72]. These studies strongly suggest that biofilm formation may plays a vital role in facilitating *H. pylori* to acquire high antibiotic tolerance; therefore, the eradication of *H. pylori* biofilm is likely a key process for clinical therapy. Nevertheless, there are challenges in clinal therapies: (1) planktonic susceptibility of minimal inhibitory concentration (MIC) may not be a reliable indicator of Minimal Biofilm Eradication Concentration (MBEC) with certain antibiotics [72,81,82]; (2) the isolation of clinical strains is not always a simple procedure as it requires the acquisition of gastric biopsies through endoscopic procedures which are not recommended as first line treatments for *H. pylori*-infected patients [11,66]. Therefore, it would be very interesting to understand if targeting biofilm formation would enhance *H. pylori* treatment.

## 8. Regulation in *H. pylori* Biofilm

Accumulating evidence suggests that *H. pylori* biofilm formation is under complicated regulations. It includes the small molecules-mediated signaling, such as AI-2 induced quorum sensing [22] and (p)ppGpp-mediated stringent response [83], two component systems, such as ArsRS acid response system [19,40,50], and transcriptional re-programing [84]. For example, dysfunction of autoinducer molecule AI-2 secretion coding gene *luxS* lead to the more robust biofilm, indicating that quorum sensing plays a regulatory role in biofilms [22]. Similarly, increased (p)ppGpp production and transcriptional upregulation of its coding gene *spoT* was both found in *H. pylori* biofilm. In turn, the absence of *spoT* results in a biofilm defect, indicating that (p)ppGpp-mediated stringent response may play an important role in regulating *H. pylori* biofilm formation [83]. In addition, mutations in the ArsRS acid response system also leads to hyper biofilm formation. In *H. pylori*, biofilm formation has also been suggested to regulations of several transcriptional regulators, such as *fliA*, *flgR*, *hp1021*, *fur*, *nikR*, and *crdR* [84].

## 9. Antibiotic Susceptibility Assessment Methods: Bacterial Viability-Based vs. Molecular-Based Techniques

To well serve for diagnostic and treatment purpose, several approaches have been commonly applied to examine *H. pylori* antibiotic susceptibility. Currently, two major types of techniques are utilized, either bacterial viability-based or molecular-based technique. Each type exhibits certain advantages and disadvantages.

Bacterial viability-based techniques are the standard approach to determine bacterial antibiotic susceptibility and has been utilized to track increasing antibiotic resistance [72,85], by measuring bacterial viability under exposure to a certain type and amount of antibiotic. Such approaches are further divided into agar or broth dilution methods, the Epsilometer test (E-test) methods [86], or disk diffusion methods [87]. These techniques are all capable of quantitatively determining the minimum concentration of an antibiotic that kills *H. pylori* [88]. Different methods have specific advantages. E-tests and disk diffusion assays are not a ‘one size fits all’ approach since the differences in susceptibility to amoxicillin, tetracycline, and furazolidone were observed between the disk diffusion method and E-test method [87]. For example, the E-test method is easy to apply and time friendly [85], while the *H. pylori* dilution method allows several stains to be tested simultaneously. However, it is noteworthy that the bacterial viability-based techniques employed planktonic cells, whose results do not naturally reflect the profile of biofilm cells.

As various antibiotic resistance mechanisms have been characterized, and the genetic elements have been identified, these discoveries promote to detect the presence of responsible antibiotic-resistant elements or susceptible elements in *H. pylori* by using molecular-based approaches [66,89,90,91]. The PCR-based genetic amplification technique and Sanger sequencing approaches together are intensively developed and applied to achieve such goals; these approaches have several advantages, including being easily reproducible and time efficient in comparison with traditional bacterial viability-based methods [92]. More importantly, these techniques can be applied directly on bacteria that have not been cultured or are at low abundance, such as various clinical isolates [93,94,95,96]. However, this approach has limitations because it is only reliable to predict certain types of antibiotics whose resistant mechanism has been specifically characterized, such as clarithromycin and tetracycline, but not for those antibiotics whose anti-mechanism is not clear yet, such as metronidazole and amoxicillin. To overcome such limitations, next generation sequencing (NGS) technologies have been developed as an efficient tool to detect and predict all potential antibiotic resistance mutations in a bacterial sample [97]. This type of approach consists of DNA extraction from a given bacterial sample that undergoes whole genome sequencing (WGS) [98]. There are several advantages of this approach compared with the PCR-based molecular approach. With the growing of whole microbial genome data sets, a pan-genome-based machine learning approach was recently developed to predict antimicrobial resistance activities in some bacteria, including *Escherichia coli* [99]. This approach uses written algorithms to predict whether a specific stain is resistant to antibiotic drugs by comparing its genome against the accessory part of the pan-genome, to yield the gene clusters that are most crucial to antimicrobial resistance activities in *E. coli*. A limitation of this approach is that we may not yet know all antibiotic resistance alleles. Currently, this approach has not yet applied in examining *H. pylori*, but it seems to be a promising one.

## 10. Mechanisms of *H. pylori* Biofilm-Promoted Antibiotic Resistance

Biofilm formation may play a significant role in facilitating *H. pylori* antibiotic tolerance [100]. A phenotype of tolerance manifests in that the antibiotic MIC for planktonic *H. pylori* does not accurately reflect the concentration needed to eradicate *H. pylori* biofilm cells. For example, a clinical study compared antibiotic susceptibility of *H. pylori* isolates between the planktonic and biofilm growth and found that *H. pylori* biofilms was more capable of tolerating various antibiotics relative to planktonic *H. pylori*, including up to 1000-fold with amoxicillin, 31.25-fold with clarithromycin, 16-fold with levofloxacin, and 8-fold with metronidazole [72]. *H. pylori* biofilms have exhibited several advantages in facilitating antibiotic tolerance. Studies have proposed the correlation between high biofilm formation capacity in *H. pylori* and the tolerance to clarithromycin, but not however, metronidazole or levofloxacin [60]. While the reason for the high tolerance of *H. pylori* biofilms is not yet fully understood, several ideas have been proposed, including that bacterial cells are protected by the biofilm structure; conjugated bacterial cells within the biofilm increase the chance of genetic exchange. Below we dissect recent mechanisms of antibiotic tolerance employed by *H. pylori* biofilms.

## 11. Extracellular Polymeric Substance Matrix Reduces the Efficacy of Antibiotics

*H. pylori* biofilms are encased in an extracellular polymeric substance (EPS) matrix that maintains the structural integrity of the biofilm, promotes adhesion, and facilitates cell-to-cell interactions [21]. Proteins, polysaccharides, and eDNA were confirmed to compose the extracellular polymeric substance matrix in *H. pylori* biofilms [25]. Immunofluorescence assays with probes specific for proteins, eDNA, and polysaccharides show that EPS distribution depends on cell density within the biofilm [19]. Polysaccharides in the EPS can be stained with FITC-conA which targets mannose groups in polysaccharides. The green fluorescence can be used to visualize the EPS matrix in *H. pylori* biofilms with Confocal Laser Scanning Microscopy (CLSM) [21,55]. The film tracer Sypro Ruby stain targets proteins in the EPS and can also be visualized using CLSM [19,25]. EPS eDNA in *H. pylori* biofilms can be stained and visualized via CLSM using BOBO-3 [25] and propidium iodide [19]. Enzymatic assays indicate that proteins play a vital role in *H. pylori* EPS as proteinase K treatment significantly causes dispersion of *H. pylori* biofilms and reduces antibiotic tolerance [19,25]. While eDNA and polysaccharides also compose EPS structures, they are predicted to play minor roles compared with proteins, based on the observation that DNase I and sodium periodate treatment targeting the eDNA and polysaccharide respectively, did not cause significant *H. pylori* biofilm reduction [19,25].

In addition to sustaining structural integrity, the EPS may reduce the efficacy of drugs from reaching the interior of the biofilm. EPS itself is minimally affected during antibiotic exposure [21], supporting the idea that antibiotic treatment does not eradicate *H. pylori* biofilms. Removal of proteins, however, does sensitize *H. pylori* in biofilms to clarithromycin, although it was not demonstrated whether this is EPS or surface protein removal [101]. Therefore, the disruption of EPS of *H. pylori* biofilm may be a highly significant target to effectively eradicate this bacterium [21].

## 12. Coccoid Cellular Morphology

Compared with the spiral shape that is commonly observed in planktonic *H. pylori* cells, coccoid cells are more commonly found in *H*. *pylori* biofilm [23,25]. The coccoid cellular shape was recognized to be the dormant state of *H. pylori* that contributes to antibiotic resistance and disease induction [58,102]. *H. pylori* biofilms, like other bacteria, can sustain the slow growth state [25], and promote antibiotic tolerance that specifically target active phase bacterium [103,104]. Prior research has shown that significant cell wall alterations occur when *H. pylori* is transitioning to the coccoid morphology [105] and has been associated with biofilm growth and antibiotic tolerance [102].

A couple of genes that modify *H. pylori’s* cell wall have been documented to be upregulated in *H. pylori* biofilms and may contribute to the coccoid form and/or antibiotic tolerance. For example, UppS, a putative undecaprenyl pyrophosphate synthase, facilitates *H. pylori* cell wall peptidoglycan modification [106]. Transposon inserted of *uppS*, resulted in a defective biofilm formation [23]. Some naturally occurring cell-wall-related mutations may be beneficial for developing antibiotic resistance. For example, recent studies found ethoxzolamide, the clinically used sulfonamide drug, can block cell wall synthesis by competitively inhibiting UppS [107]; however, strains can be become resistant by acquiring mutations in the binding site of UppS [108].

Another cell wall factor found to be important for maintaining *H. pylori* biofilm structure is peptidoglycan deacetylase (PdgA). The *pgda* gene was upregulated in *H. pylori* biofilms [25], and was previously associated with host-derived oxidative stress [109]. Oxidative stress induces *H. pylori* biofilm formation [110], which is consistent with a model that PdgA promotes *H. pylori* biofilm formation. In addition, PdgA may play an important role in maintaining *H. pylori* biofilm structure as the *H. pylori* Δ*pgdA* mutant is more susceptible to lysozyme exposure, an enzyme that cleaves the peptidoglycan of the bacterial cell wall [111].

In addition, another gene *hp0421*, encoding cholesteryl-α-glucoside transferase, was also found to regulate cellular morphology in biofilms [112,113]. The *hp0421* deletion caused defects in maintaining spiral morphology, an increase in susceptibility to antibiotics and promoted cellular aggregation to form pronounced biofilms faster than the wild-type controls [113] further supporting the important role of coccoid morphology in biofilms. In conclusion, genes that have been implicated in regulating *H. pylori* morphologies and are synchronous with affecting biofilm phenotypes and antibiotic tolerance reveal a key topic that should be investigated to further decode *H. pylori* biofilms.

## 13. Downregulated Metabolism in Biofilms

Growing bacterial cells are more easily targeted by certain types of antibiotics, such as ampicillin, that is selected as an essential component of triple therapy applications for *H. pylori* treatment [114]. Recently it has been revealed that *H. pylori* reduces its metabolic activities in the biofilm to mitigate such detrimental effects, along with the trend shifting to coccoid cellular morphology [25]. A recent clinical study found a positive correlation between strong biofilm formers and a general decrease in metabolic rate [115]. This observation is supported by another *H. pylori* transcriptomic study that suggests biofilm cells are less metabolically active than planktonic cells due to the downregulation of metabolic genes [25]. Interestingly, *H. pylori* is also able to upregulate specific metabolic enzymes to resist certain natural substrates, functionally as antibiotics. For example, Combretum mole extracts, an acetone-containing plant commonly consumed in South Africa to alleviate gastric illness, have bactericidal effects on *H. pylori* [116]. To tolerate acetone exposure, acetone carboxylase gene *acxA* is upregulated in the *H. pylori* biofilm, indicating the acetone carboxylases is expressed to potentially degrade acetone during gastric colonization. Additionally, *acxA* deletion resulted in a significant biofilm defect [23]; the *acxA* gene is under regulation of both two-component system under the ArsRS [117] and the CrdRS [118], which are heavily involved in maintaining *H. pylori* biofilm and promoting gastric gland colonization [23,119]. Both *crdR* and *arsR* regulators were found to be upregulated in biofilms [25,84,120]; *crdR* was found to be upregulated in biofilms on abiotic surfaces [25,119] and upon adherence to AGS cells [84]. On the other hand, *arsR* was found to be upregulated in strain 26695 biofilms grown on abiotic surfaces and AGS [84]. These combined findings suggest that the *acxA* gene is mandatorily expressed and essential to maintain certain functions of *H. pylori* biofilm including protecting *H. pylori* in the host from acetone degradation.

## 14. Efflux Pumps Involved Drug External Transportation

Efflux pumps are commonly located on the *H. pylori* cell membrane and facilitate the multiple drugs external transportation [121]. Efflux pumps have been strongly associated with antibiotic-resistant strains and multidrug resistance in recent studies [11,82,122] which indicates that they play a significant role in the antibiotic tolerance of *H. pylori* biofilms. Several efflux pumps coding genes, including Hp605 (*hefA*), Hp971 (*hefD*), Hp1327 (*hefG*), Hp1489, Hp1118, Hp1174 (*gluP*), HP0939, HP0497, and HP0471 (KefB), were found to be expressed in both planktonic and biofilm cells, suggesting that efflux pump is essential during *H. pylori* life cycles [83,122,123]. Recent studies further revealed that these efflux pump coding genes were significantly upregulated in biofilm to facilitate *H. pylori* antibiotics tolerance [83]. HPG27_715 (a MATE-family uncharacterized efflux pump), Hp1118, *gluP*, HP1165 (associated with tetracycline resistance), and *hefA* were significantly upregulated in biofilms relative to planktonic cells [23,83,122]. *hefA* [80,121], *hefD*, *hefG*, and HP1489 were found to be particularly upregulated in biofilms from a clarithromycin-resistant strain TK1402 [122]. *gluP* expression was found to be regulated by *H. pylori* stringent response and genetic deletions of *gluP* cause a biofilm defect and increased susceptibility to different types of antibiotics [83]. Additionally, genetic deletions in HP0939, HP0497, and KefB also conferred with a biofilm defect [123]. *H. pylori* strains isolated from Nigeria while no association with *hefG* was detected [124]. Cumulatively, *hefD* and *hefA* have both recently been associated with multidrug resistance in clinical, these findings support the perspective that *H. pylori* utilizes biofilm growth to survive under antibiotic exposure and efflux pumps are a key contributor.

## 15. Anti-Biofilm Strategies

Since chronic infection with *H. pylori* causes various gastric diseases, approaches are being developed to efficiently eradicate this bacterium. Here, we summarize several approaches based on the anti-biofilm treatments including synthetic compounds, natural compounds, and small molecule drugs.

## 16. Antimicrobial Peptides

Antimicrobial peptides (AMPs) are promising alternatives to antibiotics for combating biofilm infections. One of the advantages of using AMPs is that these molecules are also less likely to induce resistance in bacteria than antibiotics because they target multiple components within the bacterial cell. These small peptides can penetrate the extracellular matrix that surrounds biofilm cells and thus target the bacteria directly.

Another antimicrobial peptide was also recently investigated, Cbf-K16, the Cathelicidin-like peptide showed good antimicrobial activity against clarithromycin- and amoxicillin-resistant *H. pylori* in vitro and in vivo [125]. In a mouse gastritis model, Cbf-K16 demonstrated a 3.9-log_10_ reduction in bacterial counts in stomach tissues compared with an untreated mice group [125]. Interestingly, treatments with Cbf-K16 significantly downregulated the expression levels of the adhesion-associated genes *alpA* and *alpB* mRNA, both factors play a role in *H. pylori* adhesion and biofilms as mentioned above [37,38,125].

The antimicrobial peptide MSI-78A, also known as Pexiganan, is a 22-amino acid peptide Magainin-2 analogue, and was reported to have antibacterial activity in solution [125,126]. When surface-grafted, MSI_78A demonstrated activity with a high bacterial eradication rate (>90% after 2 h) thus was not able to proliferate and establish biofilms [127].

Several synthetic peptides were also applied and have been shown to promote biofilm dispersion in *H. pylori*, individually or synergistically with host antimicrobial peptides [19]. For instance, when *H. pylori* biofilms were treated with synthetic peptides IDR-1018 and DJK-5, it became more susceptible to the host-derived anti-microbial peptides [19]. In addition, DJK-5 is a synthetic short D-enantiomeric peptide designed to be resistant to bacterial proteases [128] and IDR-1018 was designed by altering bactenecin from bovine neutrophils [129]. Both DJK-5 and IDR-1018 are capable of degrading a second messenger nucleotide, a stringent response molecule, called (p)ppGpp [128,129]. Prior in vitro studies from several *H. pylori* strains (J99, 26695 and G27) suggested that *H. pylori* utilizes a stringent response at low pH or with poor nutrients to produce significant amounts of ppGpp [130]. *H. pylori* contains an enzyme called SpoT, a (p)ppGpp synthase, and hydrolase, whose genetic deletion causes a defective biofilm phenotype and an increased susceptibility to antibiotics [83]. DJK-5 and IDR-1018 were tested on *H. pylori* biofilms and were observed to not affect viability of planktonic bacterial viability; biofilm assembly, however, was inhibited only by DJK-5 (dose-dependent). In contrast, IDR-1018 reduced mature *H. pylori* biofilms without affecting the bacterial viability within the biofilm matrix [19]. These findings suggest that synthetic cationic peptides specifically target *H. pylori* in the form of biofilms and that *H. pylori* utilizes mechanisms in biofilms homologous to other bacterial species affected by the same peptides [19].

## 17. Extracts from Natural Resources

Extractions from natural resources such as plants and other bacteria are commonly applied to treat various microbial infections, including *H. pylori*. Some extractions have been found to be particularly effective in eradicating *H. pylori* by specifically targeting biofilm stability.

Probiotics can inhibit bacterial biofilms and thus play an auxiliary role in bacterial antibiotic therapy. As documented, the effects of different probiotic strains may play a varied role in restricting certain bacterial biofilms, including *H. pylori* biofilm. Probiotic Lactobacillus fermentum UCO-979C was previously found to play a role in inhibiting *H. pylori* biofilm formation [131]. Furthermore, another microbial study found that *Lactobacillus plantarum* LN66 cell-free supernatant (CFS) can weaken *H. pylori* biofilm formation, an effect monitored by SEM and confocal laser scanning microscopy (CLSM) [132]. Probiotics combined with other antibiotics were found to increase treatment efficacy for levofloxacin as LN66 CFS facilitates this antibiotic function to inhibit EPS secretion [131,133]. Another intriguing finding is armeniaspirols, which is a novel class of natural products isolated from *Streptomyces armeniacus* previously identified as antibacterial agents against Gram-positive pathogens [134]. Armeniaspirol A (ARM1) exhibited potent antibacterial activity against *H. pylori* as well by inhibiting *H. pylori* biofilm formation in a dose-dependent manner. In a mouse model to study multidrug-resistant *H. pylori*, dual therapy with ARM1 and omeprazole showed efficient killing efficacy, comparable to the standard triple therapy, and induced negligible toxicity against normal tissues [135]. Moreover, at acidic pH 2.5, ARM1 exhibited a much more potent anti-*H. pylori* activity than metronidazole [135]. All these advantages promote the possibility of ARM1 being used in a clinical application.

Extracted organic products from plants are also important to treat bacterial infections. A variety of materials have been found to efficiently restrict *H. pylori* infection. For example, *Antractylodes lancea* volatile oils were recently found to inhibit *H. pylori* biofilm formation. This oil complex also exhibits a robust ability to reduce *H. pylori* virulence factor CagA translocation into host cells, a finding observed in a cell culture infection model [136]. Additional screenings were applied to search for natural molecules to target *H. pylori* biofilm stability. Phytochemicals from *Acorus calamus*, *Colocasia esculenta Vitex trifolia*, *Azadirachta indica* A. *Juss* exhibited a significant effect on inhibiting *H. pylori* biofilm formation as well [137,138]. Among screening tests, *Acorus calamus* exhibited the highest *H. pylori* anti-biofilm activity via a dose-dependent pattern [138]. Phytochemicals from the neem tree (*Azadirachta indica* A. Juss) were also previously shown to have bactericidal properties and several other Neem tree phytochemicals (nimbolide, azadirachtin, and gedunin) and were tested for toxicity towards *H. pylori* but only Nimbolide was found to kill both planktonic and biofilm *H. pylori* without having hemolytic activity; Nimbolide was effective towards the nine strains of *H. pylori* tested in a time and dose-dependent manner under various stressful growth conditions and metabolic activities [137]. Dihydroatanshinone I, a natural herbal compound, is another agent that clearly inhibits *H. pylori* biofilm in both in vitro and in vivo studies when combined with omeprazole as a dual therapy, even more efficiently compared with the standard triple therapy approach; more interestingly, this compound exhibited negligible toxicity against normal tissues, indicating the potential in its clinical application [57]. Extracts from hibiscus flowers (*Hibiscus rosa sinensis L. flower*) also showed properties of inhibiting biofilms and bactericidal effects on drug-resistant *H. pylori* strains [139]. Alginate lyases, a compound found naturally in brown algae that degrades the EPS, was found to enhance the efficacy of clarithromycin when both components are synergistically used to treat biofilms [31].

These recent findings open exciting possibilities for discovering natural compounds that effectively target and eliminate *H. pylori*, even in biofilm forms. Although not all these drugs have been tested in in vivo conditions, it is essential to investigate their potential as anti-*H. pylori* agents given that their effectiveness has already been confirmed in vitro.

## 18. Small Molecule Drug and Nanodrugs

Various small molecule-based compounds that facilitate traditionally applied antibiotics, have been found to be effective at treating bacterial infections. These compounds include both organic and inorganic monomers or polymers that target bacterial essential enzymes, pathways, or structure. For example, carvacrol and thymol were found to inhibit *H. pylori* biofilms by inhibiting an enzyme required for biofilm growth, carbonic anhydrase [140]. Lipid polymer nanoparticles can eradicate *H. pylori* biofilm by enhancing the encapsulation of a given antibiotic, such as clarithromycin, to reduce biofilm viability and structural integrity more efficiently via bypassing the mucus layer and the EPS of the *H. pylori* biofilm [21]. A following study further found that the function of *N*-acylhomoserine lactonase silver nanoparticles (aka nanodrugs) in inhibiting *H. pylori* quorum-sensing system, potentially combats *H. pylori* biofilm formation [141]. Additionally, synthesized silver ultra-nano clusters (SUNCs) in another study were found to inhibit *H. pylori* biofilm formation when synergized with other antibiotics, like metronidazole [142,143]. Nanodrugs are slightly negative-charged/ hydrophilic oral drugs fabricated of berberine derivatives and rhamnolipids (RHL) that penetrate the mucus layer and effectively clear *H. pylori* biofilms in vitro and in vivo [21,55]; RHL is a biosurfactant composed of di and mono-rhamnose sugars attached to fatty acids produced by *Pseudomonas aeruginosa* [21] and berberine is a quaternary ammonium alkaloid isolate from *Coptis chinensis* that is proposed to enhance the efficacy of triple therapy for *H. pylori* infections [55]. Nanoparticles modified with mannose were specifically found to be effective towards multi-drug-resistant *H. pylori* and their biofilms [144]. All these studies show that the combination of nanodrugs with antibiotics efficiently disrupts *H. pylori* biofilm and provides a feasible strategy to eradicate *H. pylori* infection.

## 19. Conclusions and Perspective

In conclusion, the scientific community has made considerable strides in unraveling the intricate nature of the gastric chronic pathogen, *H. pylori*, and its biofilm formation mechanisms. Notably, studies employing clinically isolated strains have played a crucial role in advancing our understanding and have paved the way for the development of promising biofilm-based approaches for eradicating *H. pylori*.

Nevertheless, the complexity of in vivo environment and the limitation of current developed techniques cause difficulty of characterizing in vivo bacterial biofilm, including *H. pylori*, and studying the effect of treatment candidates. Future studies are required to comprehensively evaluate the efficiency of recently proposed treatments on *H. pylori* eradication. These investigations are expected to extend beyond in vitro experiments and encompass comprehensive animal models and rigorous clinical trials. By conducting such studies, we can obtain a more accurate assessment of the therapeutic potential of these proposed treatments and their impact on both the host and the pathogen.

However, the complexity of the in vivo environment and the limitations of current developed techniques make it challenging to characterize bacterial biofilm within host, including *H. pylori*, and study the effects of treatment candidates. Future studies will need to go beyond in vitro experiments and incorporate comprehensive animal models and rigorous clinical trials. Through conducting such studies, we can obtain a more accurate assessment of the therapeutic potential of these proposed treatments and their impact on both the host and the pathogen.

Furthermore, it is important to explore the long-term effects of these novel approaches to ensure their safety and efficacy in real-world scenarios. Additionally, investigating potential resistance mechanisms that *H. pylori* may employ in response to biofilm-targeting therapies would be instrumental in designing more robust treatment strategies.

In conclusion, while significant progress has been made in understanding *H. pylori* biofilm formation and developing potential eradication approaches, further research is necessary to evaluate the pharmacological effects, efficacy, and safety of these treatments in animal models and clinical trials. By addressing these research gaps, we can bring us closer to achieving more effective and personalized strategies for combating *H. pylori* infection and its associated complications.

## Data Availability

Not applicable.
